# Dual-Function Adjuvant Cyclosporin A: Enhancing RSV-Specific Humoral Immunity via Treg-Driven B-Cell Activation

**DOI:** 10.3390/vaccines13100997

**Published:** 2025-09-23

**Authors:** Chaofan Li, Yiwei Zhong, Shuren Zhang, Caixia Su, Gan Zhao, Bin Wang

**Affiliations:** 1 MOE/NHC/CAMS Key Laboratory of Medical Molecular Virology, Shanghai Institute of Infectious Disease and Biosecurity, Shanghai Frontiers Science Center of Pathogenic Microorganisms and Infection, School of Basic Medical Sciences, Shanghai Medical College, Fudan University, Shanghai 200032, China; djp5yp@virginia.edu (C.L.); yiweizhong@fudan.edu.cn (Y.Z.); zhangsr@pregene.com (S.Z.); caixia.su@yitherbiotech.com (C.S.); 2 Advaccine Biopharmaceuticals (Suzhou) Co., Ltd., Suzhou 215000, China; zhaog@advaccine.com; 3 Children’s Hospital, Fudan University, Shanghai 201102, China

**Keywords:** RSV vaccine, cyclosporin A, Treg cells, B-cell activation, antibody production, adjuvant, immunomodulation

## Abstract

Background: Respiratory syncytial virus (RSV) remains a leading cause of respiratory illness globally, with limited vaccine options, particularly for infants and high-risk populations. This study investigates Cyclosporin A (CsA), traditionally an immunosuppressant, as a novel adjuvant to enhance RSV-specific immunity. Methods: BALB/c mice were subcutaneously immunized with RSV G protein co-administered with varying Cyclosporin A doses, challenged intranasally with RSV, and analyzed for RSV-specific humoral immunity and mechanistic Treg-dependent B-cell responses. Results: We demonstrate that co-administration of CsA with the RSV G protein (G+CsA) dose-dependently boosts RSV-specific IgG and neutralizing antibodies, with selective augmentation of IgG1 and IgG2 subclasses. Mechanistically, G+CsA induces regulatory T cells (Tregs) expressing CD40L and IL-10, which directly promote B-cell activation, proliferation, and plasma cell differentiation. Depletion of Tregs or neutralization of IL-10/CD40L abrogated antibody production, confirming these pathways as critical mediators. Notably, G+CsA-induced Tregs adopt a helper phenotype distinct from conventional Tregs, balancing immune enhancement and homeostasis. Conclusions: CsA demonstrates dual adjuvant properties by enhancing RSV-specific neutralizing IgG titers through Treg-driven B-cell activation, offering a potential strategy to optimize vaccine-induced humoral immunity.

## 1. Introduction

Respiratory syncytial virus (RSV) is a leading cause of respiratory illness globally, annually affecting millions in the United States alone [1,2]. Severe RSV infections hospitalize ~57,000 children under five and cause ~14,000 deaths in older adults annually [3], with economic costs exceeding USD 1 billion/year [4,5]. Transmission via respiratory droplets and close contact (e.g., daycare centers, hospitals) amplifies spread [6,7], while prematurity, chronic lung disease, and immunodeficiency exacerbate severity [8,9,10].

Despite urgent need, licensed RSV vaccines are limited. Three recent approvals—Arexvy (GSK), Abrysvo (Pfizer), and mRESVIA (Moderna)—show efficacy in older adults. Notably, Abrysvo has shown promise in its ability to protect newborns by allowing vaccinated pregnant individuals to transfer antibodies via the placenta [2,11]. Additionally, while Nirsevimab and Palivizumab have been administered to infants and young children [12,13], no vaccines are specifically approved for infants/young children, who bear the highest morbidity/mortality [14,15,16,17]. Vaccine development for this vulnerable group faces hurdles, notably vaccine-associated enhanced disease (VAERD) linked to the 1960s FI-RSV vaccine [18,19,20,21]. VAERD pathogenesis involves multifactorial mechanisms, including inadequate neutralizing antibody induction, Th2-polarized CD4+ T-cell responses, impaired interferon signaling, and pulmonary eosinophilic inflammation [22]. For example, Th2-skewed immunity exacerbates airway pathology in RSV-challenged mice [18], while IgA deficiency correlates with enhanced disease severity in infants [23]. Therefore, the development of RSV vaccines for infants and young children has faced significant challenges. While recent efforts explored novel platforms like mRNA and viral vectors to mitigate VAERD risks, these approaches encountered setbacks: mRNA-based RSV vaccines failed in pediatric trials due to severe VAERD and mortality [24]. The phase 1 trial of Moderna’s mRNA-1345 and mRNA-1365 vaccines in infants and toddlers was stopped by the FDA due to safety concerns. The trial identified an imbalance in rates of severe respiratory events, suggesting a potential risk of VAERD. Viral vector candidates underperformed in older adults [25]. These outcomes underscore the need for alternative strategies. The current research now prioritizes live-attenuated formulations, subunit vaccines targeting conserved viral epitopes, or immunomodulatory adjuvants to balance immune activation and regulation. A key focus is harnessing regulatory T cells (Tregs) to suppress excessive inflammation while preserving protective immunity [26,27,28]. This dual approach—targeting viral components and modulating immune pathways—aims to overcome historical hurdles and deliver safe, effective vaccines for vulnerable populations.

Emerging evidence suggests Tregs can modulate B-cell function, potentially boosting antibody production [29,30,31,32]. This interplay offers opportunities to design vaccines that harness Tregs to enhance protection without VAERD risks.

Cyclosporin A (CsA), traditionally an immunosuppressant, paradoxically enhances immune responses when co-administered with RSV antigens [31,32]. Our findings indicate that protein plus a low dose of CsA immunization induces antigen-specific Tregs, which help minimize pathogenic T-cell responses and promote the production of high levels of neutralizing antibodies [32]. The combination of CsA and G protein significantly reduced VAERD as shown by histopathology, reduced BALF cellularity, and lower lung index scores, compared with G protein or CsA alone. Importantly, the expansion of Tregs appears to be mediated through a CsA-induced tolerogenic mechanism that modulates dendritic cell (DC) function. Specifically, CsA interferes with DC maturation, preventing its full activation and promoting a semi-mature or tolerogenic DC phenotype [33]. This duality suggests CsA engages complex, incompletely understood immunomodulatory pathways, particularly in regulating B-cell function.

RSV is a member of the *Pneumoviridae* family and has a single-stranded, negative-sense RNA genome. It is divided into two major antigenic subgroups, A and B. The virus has two key surface glycoproteins, the G protein (attachment) and the F protein (fusion), which are the primary targets for neutralizing antibodies. Historically, the G protein has received less attention in vaccine development due to its linear structure and mucin-like properties. However, recent progress has highlighted its potential, as several high-affinity neutralizing monoclonal antibodies against G protein—such as 131-2G and 3D3—have been successfully developed against RSV [34,35], underscoring its viability as a target for future therapies [34]. Here, we investigated CsA’s role as an RSV G protein adjuvant, focusing on how CsA-induced regulatory T cells (Tregs) drive B-cell activation and plasma cell differentiation. Our results show that G+CsA immunization significantly boosts RSV-specific IgG and neutralizing antibodies. Mechanistically, CsA-induced Tregs—marked by elevated CD40L and IL-10 expression—promote B-cell proliferation and antibody production. These findings reveal CsA’s dual capacity as an immunosuppressant and immune enhancer, supporting its potential as a vaccine adjuvant to optimize RSV-specific humoral immunity.

## 2. Materials and Methods

### 2.1. Animals, Virus, and Infection

Female 6- to 10-week-old BALB/c mice were procured from the Shanghai SLAC Laboratory Animal Co., Ltd. (Shanghai, China). Foxp3-DTR/EGFP mice on a BALB/c background were obtained from The Jackson Laboratory (Sacramento, CA, USA). The mice were housed under specific pathogen-free conditions at Fudan University (Shanghai, China) and handled in accordance with the animal welfare guidelines for experimental animals. Protocols related to this study were reviewed and approved by the Committee of Experimental Animals of SHMC (No. 20240229-027). Plaque-purified human RSV (type A2 strain, ATCC VR-1546, from the American Type Culture Collection, Rockville, MD, USA) was propagated in HEp-2 cells (ATCC CCL-23) at an MOI of 0.01. The virus was harvested at 4.5 days post-infection and concentrated by ultracentrifugation (Optima L-100XP Ultracentrifuge, Beckman Coulter, Brea, CA, USA) at 50,000× *g* for 1 h. All groups of mice were briefly anesthetized with isoflurane gas (Rotho Pharmaceuticals, Shanghai, China) before being intranasally infected with 5 × 10^7^ plaque-forming units (PFUs) RSV in 100 µL on day 28 after the second immunization. The RSV challenge dose (5 × 10^7^ PFU) mirrors established models of severe infection [36]. Heat-attenuated RSV retains conformational epitopes and was used for the ELISA.

### 2.2. Vaccine Preparations and Immunization

The RSV attachment glycoprotein (G protein) extracellular domain (amino acids 67–298) was cloned into the pET28a plasmid using restriction sites for NcoI and XhoI. The construct was transformed into *Escherichia coli* BL21 (DE3) (New England Biolabs, Ipswich, MA, USA). G protein was chromatographically purified and lyophilized by Beijing Advaccine Biotech Co., Ltd. (Beijing, China). cGMP-grade Cyclosporin A (Batch No. 20230101, SANTAI Pharmaceutical Co., Ltd., Taishan, China) was formulated in Kolliphor HS 15 (BASF, Ludwigshafen, Germany) at 0.1 mg/mL as a diluent. Lyophilized and purified G protein was reconstituted with 100 µL of CsA-containing diluent at serial dilutions before immunization. Mice were randomly assigned to experimental groups and subcutaneously (s.c.) immunized on the right side of the back on days 0 and 14 with 10 µg of G protein, CsA alone, or 10 µg of G protein with 1, 2, 4, 6, 8, or 10 µg of CsA. The vaccination volume per mouse was 100 µL [32,37].

All formulations, including G protein-only controls, contained equivalent concentrations of Kolliphor HS15. Endotoxin levels in recombinant RSV G protein were confirmed via LAL assay to be <5 EU/mL (Supplementary Table S1). The prime/boost intervals (14 days) align with murine vaccine protocols optimizing GC responses [38].

### 2.3. Enzyme-Linked Immunosorbent Assay (ELISA)

Serum samples from immunized mice were collected on day 28. ELISA was used to quantify RSV-specific IgG, IgG1, IgG2a, IgG2b, and IgG3 titers in serum as previously described [39]. Briefly, 96-well plates (Corning, NY, USA) were coated with 5 × 10^6^ PFU/mL heat-inactivated RSV (50 mM carbonate bicarbonate buffer, pH 9.6) at 37 °C and blocked with 5% BSA in PBST (0.05% Tween 20 in PBS) at 37 °C. Plates were incubated with serial 2-fold dilutions of serum for 1 h at 37 °C. HRP-conjugated secondary antibodies (Southern Biotech, Birmingham, AL, USA) were applied and incubated for 45 min at 37 °C.

### 2.4. Viral Neutralizing Antibody Assay

The neutralizing antibody assay was performed as previously described [32]. Serum samples were serially diluted 5-fold in PBS, heat-inactivated at 56 °C for 30 min, and incubated with a 3 × 10^3^ 50% tissue culture infective dose virus for 2 h at 4 °C. HEp-2 cells (ATCC CCL-23) were added to each well. Plates were incubated for 3 days in a 5% CO_2_ incubator at 37 °C, fixed with 80% cold acetone in PBS, and blocked with 3% blocking buffer. HRP-Goat anti-RSV antibody (Meridian Life Science, Saco, ME, USA) was added, and the enzymatic reaction with 3,3′,5,5′-tetramethylbenzidine (TMB) (Beyotime, Shanghai, China) was developed. Optical densities were read at 450/620 nm using an ELISA plate reader (Thermo Fisher Scientific, Waltham, MA, USA). The Reed–Muench method was selected for the neutralizing antibody titers’ efficiency in calculating 50% neutralization endpoints (NTID50) in high-throughput settings.

### 2.5. Pulmonary Lymph Nodes and Spleen Isolation and Flow Cytometry

Mice were sacrificed using CO_2_ on day 32 to provide a 4-day period after Treg reactivation induced by G protein or RSV challenge on day 28, thereby enriching antigen-specific recalled Tregs. Lung lymph nodes and spleens were then collected for analysis. Single-cell suspensions were prepared through a 40 µm strainer (BD Falcon, Franklin Lakes, NJ, USA). Briefly, 5 × 10^5^ to 1 × 10^6^ cells per sample for lung lymph nodes, and 1 × 10^6^ cells per sample for spleens were used for FACS analysis. For B-cell and plasma cell analysis, cells were stained with viability marker Fixable Viability Dye eFluor 780 (eBioscience, San Diego, CA, USA) and the following antibodies: anti-CD19-BV421 (Biolegend, San Diego, CA, USA), anti-B220-BV605 (Biolegend), anti-GL7-PE (eBioscience), anti-CD95-APC (Biolegend), and anti-CD138-BV421 (Biolegend). For Treg and cytokine analysis, cells were stained with Fixable Viability Dye eFluor 780 and the following antibodies: anti-CD3-BV421 (Biolegend), anti-CD4-BV605 (Biolegend), anti-Foxp3-FITC (Biolegend), anti-CD40L-BV510 (Biolegend), anti-CD25-PE (eBioscience), anti-IL-10-PerCP-Cy5.5 (eBioscience), and Foxp3 fixation/permeabilization kit (eBioscience). Data were acquired using LSRFortessa (BD Biosciences, San Jose, CA, USA) and analyzed with FlowJo software v10.8.1 (BD Biosciences).

### 2.6. Treg Depletion

Diphtheria toxin (DT) (Sigma-Aldrich, St. Louis, MO, USA) was suspended in PBS at 5 µg/mL. Foxp3-DTR/EGFP mice were injected intraperitoneally with 500 ng DT in 100 µL PBS on days -2 and -1 before the first immunization and again on days 12 and 13 before the second immunization. The presence of Foxp3+ in CD3+CD4+ T cells in peripheral blood was examined every two days from day 0 until Foxp3 levels recovered.

### 2.7. Treg, Tcon, and B-Cell Isolation and Co-Culture Assay

Splenocytes were isolated from mice sacrificed using CO_2_ on day 32. Treg and Tcon cell populations were identified by flow cytometry using viability marker Fixable Viability Dye eFluor 780 (eBioscience) and the following antibodies: anti-CD3-FITC (Biolegend), anti-CD4-eFluor 450 (Biolegend), and anti-CD25-PerCP-Cy5.5 (Biolegend). GC B cells were identified using viability marker Fixable Viability Dye eFluor 780 (eBioscience), anti-B220-APC (Biolegend), and anti-GL7-PE (Biolegend) antibodies. FACSAria II (BD Biosciences) was used to sort eFluor 780-CD3+CD4+CD25+ Tregs, eFluor 780-CD3+CD4+CD25- Tcon cells, and CD220+GL7+ GC B cells. GC B cells (5 × 10^4^ cells) were co-cultured with Treg (1 × 10^6^ cells) or Tcon cells (1 × 10^6^ cells) and stimulated with heat-inactivated RSV at 37 °C/5% CO_2_ for 3 days. Supernatants were collected and analyzed for total IgG and IL-10 using a cytometric bead array flex set (BD Biosciences). GC B-cell proliferation was detected by intracellular staining with anti-Ki67 antibody (eBioscience).

### 2.8. IL-10 and CD40L Blockade In Vitro and In Vivo

For in vitro antibody blocking experiments, co-culture systems were treated with blocking antibodies: 5 µg/mL anti-CD40L (InVivoMAb, Durham, NC, USA) and 5 µg/mL purified anti-IL-10 (InVivoMAb) or isotype rat IgG1 (InVivoMAb). For in vivo antibody blocking, mice were administered intraperitoneally with 100 µg in 100 µL per mouse of purified anti-CD40L, purified anti-IL-10, or isotype rat IgG1 one day prior to each immunization.

### 2.9. Statistical Analysis

Data are presented as means ± SEM. Statistical analysis was conducted using GraphPad Prism software (V9.5.1) (La Jolla, CA, USA). Differences in mean values between groups were assessed using the Student’s *t* test, One-way ANOVA or Two-way ANOVA as indicated in the figure legend. The significance levels are as follows: **** *p* < 0.0001; *** *p* < 0.001; ** *p* < 0.01; * *p* < 0.05; not significant, *p* > 0.05. Data points in all figures represent independent spleens or lymph nodes, unless otherwise specified.

## 3. Results

### 3.1. G+CsA Immunization Promotes RSV-Specific IgG Production and Isotype Switching

**Figure 1:**
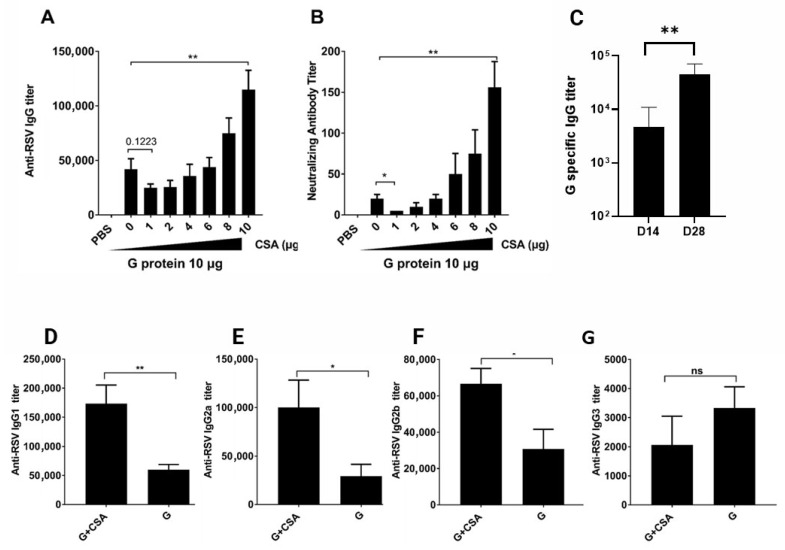
G+CSA immunization promotes antibody production. Mice were immunized subcutaneously (s.c.) with 10 μg G protein with CsA doses ranging from 0 to 10 µg on days 0 and 14; anti-RSV IgG titer (**A**) and neutralizing antibody titer (**B**) were measured on day 28. (**C**) Kinetic curve of anti-RSV IgG titer after immunization with G+CsA (10 μg + 10 μg/mouse). Subtypes of anti-RSV IgG1 (**D**), anti-RSV IgG2a (**E**), anti-RSV IgG2b (**F**), and anti-RSV IgG3 (**G**) 14 days post-2nd immunization. Data are means ± SEM from two independent experiments (*n* = 6). ** *p* < 0.01, * *p* < 0.05, ns (not significant) were determined by One-way ANOVA (**A**,**B**) or Student’s *t* test (**C**–**G**).

To evaluate CsA’s influence on humoral immunity, Balb/c mice were immunized subcutaneously with escalating doses of CsA (0–10 µg) co-formulated with a fixed 10 µg dose of recombinant RSV G protein on days 0 and 14. Serum collected on day 28 revealed dose-dependent increases in RSV-specific IgG and neutralizing antibody titers (Figure 1A,B), with 10 µg CsA significantly enhancing both metrics (*p* < 0.01 vs. 10 µg G protein alone). The neutralizing titer in the serum was enhanced 5–10-fold by CsA + G immunization. However, the binding antibody titer against G protein was only enhanced 3-fold. It suggested that the CsA+G immunization preferentially enhances the neutralizing antibody response compared with the overall binding antibody titers. Our previous findings [40] support this, showing that antibodies induced by the G+CsA combination could effectively compete with the 131-2G monoclonal antibody for binding to the central conserved domain (CCD), directly implicating the targeting of this critical CX3C region. Kinetic analysis of anti-RSV IgG levels (Figure 1C) showed a marked rise in the G+CsA group on day 28 (14 days post-second immunization), compared to the levels on day 14 (14 days post-first immunization). In the G+CsA group, RSV-G IgG levels increased 8-fold from day 14 (5600 ± 2909) to day 28 (44,800 ± 11,758). This elevation aligns with the biological timeline of B-cell maturation and class switching, which typically requires >10 days post-boost to generate a high level of IgG. An isotype-specific ELISA further demonstrated that CsA selectively augmented the IgG1 and IgG2 subclasses (Figure 1D–F), while IgG3 remained unaffected (Figure 1G). These data indicate that CsA, at an optimal dose, acts as an immunomodulator to boost antibody production and drive targeted isotype switching, potentially enhancing vaccine efficacy.

### 3.2. Effects of G+CsA Immunization on B-Cell Differentiation

**Figure 2:**
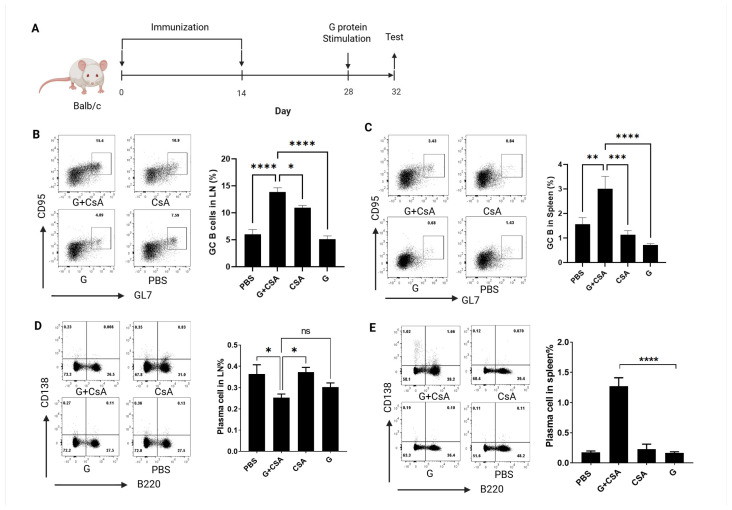
G+CsA-primed B cells exhibit a rapid and efficient response to antigen stimulation. (**A**) Schematic of immunization and antigen stimulation, mice were immunized with 10 μg G + 10 μg CsA on days 0 and 14; then G protein (10 μg/mouse) was injected s.c. on day 28 as in vivo challenge; CD19^+^B220^+^Fas^+^GL7^+^ GC B cells and CD138^+^B220^−^ plasma cells in dLNs and spleens were tested on day 32 without in vitro stimulation. GC B cell from dLNs (**B**) and spleens (**C**) were tested by flow cytometry with specific antibodies. Plasma cell population was tested on day 32 from dLNs (**D**) and spleens (**E**). Data are means ± SEM from two independent experiments (*n* = 6). **** *p* < 0.0001, *** *p* < 0.001, ** *p* < 0.01, * *p* < 0.05, ns (not significant) were determined by One-way ANOVA (**B**–**E**).

To investigate the effects of G+CsA immunization on B-cell differentiation, we utilized both GL7 and CD95 as markers for the definition of germinal center (GC) B cells. The GC is the site of robust B-cell activation and antibody affinity maturation [38,41]. Mice received two subcutaneous immunizations with RSV G protein (with or without CsA) on days 1 and 14, followed by a G protein boost on day 28. Four days post-challenge, flow cytometry analyzed B-cell subsets in draining lymph nodes (inguinal LNs (dLNs)) and spleens (Figure 2A). We observed a trend toward significantly increased CD19^+^B220^+^GL7^+^CD95^+^ GC B cells in the dLNs and spleens of G+CsA-immunized mice compared with the CsA-alone, G-alone, and PBS control groups (Supplementary Figure S1, Figure 2B,C). To further examine the differentiation of the GC B cell post-immunization, we checked the total plasma cells in the lung dLNs and spleen. CD138^+^B220^-^ cells were analyzed as plasma cells, consistent with prior characterization of CD138 (syndecan-1) as a surface marker for antibody-secreting plasma cells [42]. In the dLNs, the G+CsA immunization group did not exhibit a significant accumulation of plasma cells compared with the G protein-immunized mice or the PBS control group (Figure 2D). However, G protein single immunization did not alter the presence of plasma cells in the spleen, while the percentage of spleen plasma cells significantly increased post-G+CsA immunization (Figure 2E), which indicated the function of CSA in active plasma cell differentiation or accumulation as the adjuvant. These data indicate that CsA co-administration with G protein enhances B-cell activation in dLNs and spleens, compared with the CsA-alone and G-alone groups. The G +CsA group promotes plasma cell generation in spleens, compared with the CsA-alone, G-alone, and PBS groups, suggesting distinct tissue-specific roles in antibody production.

### 3.3. Treg Cells Are Critical for B-Cell Activation Post-G+CsA Immunization

**Figure 3:**
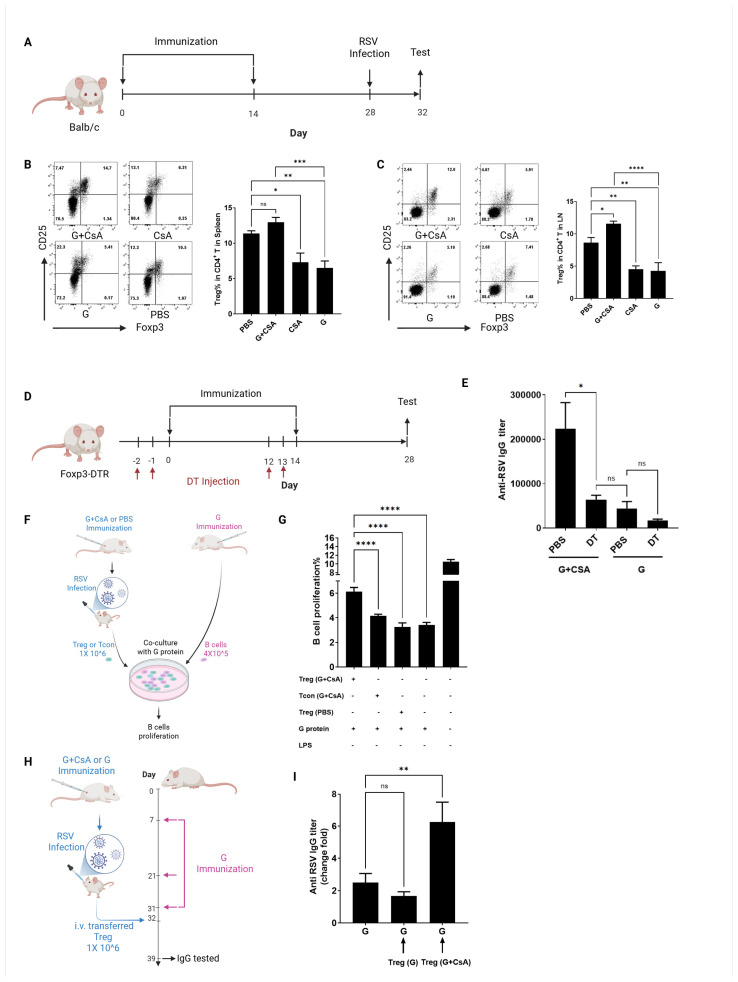
G+CsA-induced Treg cells promote B-cell activation. (**A**) Schematic of immunization and RSV challenge. Balb/c mice were immunized s.c. on days 0 and 14 with 10 μg G + 10 μg CsA, 10 μg CsA, or 10 μg G prior to the RSV challenge on day 28. The ratio of Treg cells (CD4+CD25+FoxP3+) in the spleens (**B**) and pulmonary LNs (**C**) of the infected mice were tested by FACS on day 32 without in vitro stimulation. (**D**) Schematic of Treg cell depletion. (**E**) Foxp3-DTR/EGFP mice were injected with diphtheria toxin (DT) two days each time before immunization; anti-RSV IgG titers were tested by ELISA on day 28. (**F**) Schematic of Treg cell and B-cell co-culture in vitro. G protein-primed B cells labeled with eFluor 670 were co-cultured with Treg cells or Tcon cells from G+CsA-immunized and then RSV-infected mice in the presence of G protein stimulation. Three days later, B-cell proliferation was tested on day 28. (**G**) The ratio of B-cell proliferation after co-culture with different T cells and antigens was analyzed. (**H**) Schematic of Treg cells help B-cell activation in vivo. G+CsA- or G protein-alone-induced Treg cells were adoptive-transferred into G protein immunized mice trice; anti-RSV IgG titers were tested and compared with the titer before transfer on day 39. (**I**) Levels of anti-RSV-specific IgG titers were analyzed. Data are means ± SEM from two independent experiments (*n* = 8). **** *p* < 0.0001, *** *p* < 0.001, ** *p* < 0.01, * *p* < 0.05, ns (not significant) were determined by One-way ANOVA (**B**,**C**,**E**,**G**,**I**).

To elucidate the mechanisms underlying CsA’s impact on B-cell responses, we first assessed the role of T follicular helper (Tfh) cells, which are critical for germinal center (GC) formation and antibody affinity maturation. Mice immunized with G+CsA and challenged with RSV exhibited no significant changes in splenic CD3^+^CD4^+^PD-1^+^CXCR5^+^Tfh cell frequency or IL-21 expression compared with G-only controls (Supplementary Figure S2A–C, *p* > 0.05). This suggests that the effects of CsA on B-cell activation occur independently of Tfh cells. Given prior evidence that G + CsA expands antigen-specific regulatory T cells (Tregs) [32], we hypothesized that Tregs might drive B-cell responses. Balb/c mice were immunized s.c. on days 0 and 14 with G + CsA, CsA alone, or G protein alone prior to the RSV challenge on day 28 (Figure 3A). Flow cytometry revealed a significant increase in Treg cells (CD4^+^CD25^+^FoxP3^+^) in the spleens (*p* < 0.001 vs. G alone; Figure 3B) and pulmonary LNs of G+CsA-immunized mice post-RSV challenge (*p* < 0.0001 vs. G alone; Figure 3C). To directly test Treg dependency, we utilized Foxp3-DTR/EGFP mice, which enable Treg depletion via diphtheria toxin (DT). Mice were immunized with G+CsA or G alone, with DT administered prior to each dose (Figure 3D and Supplementary Figure S3). ELISA analysis showed that DT-mediated Treg depletion significantly reduced anti-RSV IgG titers in the G+CsA group (*p* < 0.05 vs. PBS-treated controls in the G+CsA group; Figure 3E), with levels comparable to those in G-only immunized mice. This result demonstrates that Tregs are essential for G+CsA-induced B-cell activation and antibody production.

To further dissect Treg-mediated B-cell activation, we sorted CD4^+^CD25^+^FoxP3^+^ Treg cells and CD4^+^CD25^−^FoxP3^−^ conventional T cells (Tcons) from G+CsA-immunized mice post-RSV challenge. These cells were co-cultured with G protein-primed B cells in the presence of cognate antigen (Figure 3F). After 3 days, only Treg cells from G+CsA-immunized mice robustly promoted B-cell proliferation (*p* < 0.0001 vs. controls; Figure 3G), while Treg cells from PBS-treated mice or Tcons from G+CsA immunizations failed to enhance proliferation. To validate this effect in vivo, we performed adoptive transfer experiments. Treg cells isolated from G+CsA- or G-only immunized mice were transferred into G protein-primed recipients, followed by RSV challenge. Anti-RSV IgG titers were measured 7 days post-transfer (day 39). Recipients of G+CsA-derived Treg cells exhibited significantly elevated IgG titers (*p* < 0.01) compared with pre-transfer levels (day 28; Figure 3H,I), whereas Treg cells from G-only immunized mice showed no such effect. These data confirm that G+CsA-induced Treg cells directly facilitate B-cell proliferation and antibody production, highlighting their non-redundant helper function in this context.

### 3.4. Treg Cell-Derived CD40L and IL-10 in Drives B-Cell Differentiation into Plasma Cells

**Figure 4:**
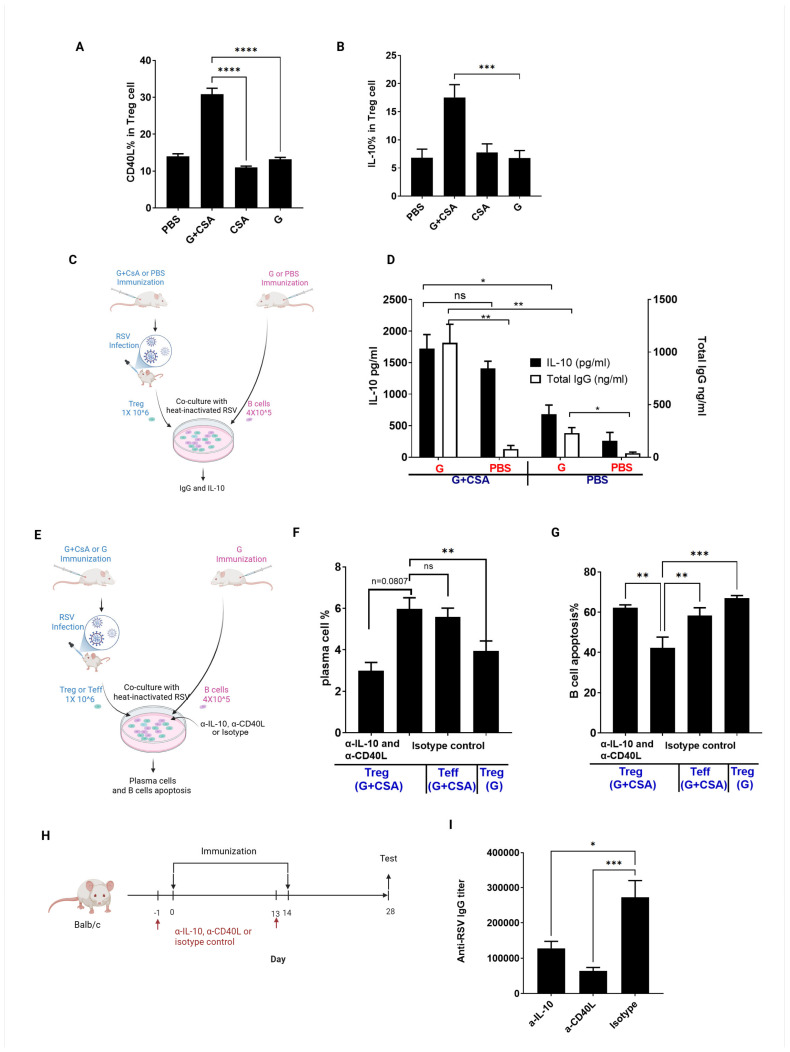
G+CsA-induced Treg cells promote B-cell differentiation via CD40L expression and IL-10 secretion. Balb/c mice were immunized s.c. on days 0 and 14 with 10 μg G + 10 μg CsA, 10 μg CsA, or 10 μg G and infected with RSV on day 28 after the 2nd immunization as illustrated in Figure 3A. The expression level of CD40L (**A**) and IL-10 (**B**) in Treg cells isolated from spleens of the infected mice were tested by FACS on day 32 without in vitro stimulation. (**C**) Schematic of IL-10 promoting antibody production in vitro. Treg cells from G+CsA- or PBS-immunized donor 1 mice were co-cultured with B cells of G protein- or PBS-immunized donor 2 mice; levels of total IgG and IL-10 in the medium were analyzed 3 days later after the co-culture. (**D**) Concentration of IL-10 and total IgG in the medium after day 3 of co-culture in vitro. (**E**) Schematic of Treg cells promote B-cell differentiation and survival in vitro. G protein-primed splenocytes were co-cultured with G+CsA-induced Treg cells; the ratio of plasma cells and apoptosis of B cells was tested 3 days later. (**F**) The ratio of plasma cell after co-cultured with different T cells and with or without α-IL-10 and α-CD40L, respectively. (**G**) The ratio of apoptotic B cells after co-culture with different T cells and with or without α-IL-10 and α-CD40L. (**H**) For the in vivo antibody blocking experiment, mice were administered intraperitoneally with 100 µg in 100 µL per mouse of α-IL-10, α-CD40L, or isotype control antibody, respectively, on days -1 and 13 before immunization. (**I**) Anti-RSV IgG titers were tested 14 days after the 2nd immunization. Data are means ± SEM from two independent experiments (*n* = 8). **** *p* < 0.0001, *** *p* < 0.001, ** *p* < 0.01, * *p* < 0.05, ns (not significant) were determined by Two-way ANOVA (**A**,**B**,**D**,**F**,**G**,**I**).

To elucidate the mechanisms by which G+CsA-induced Treg cells promote B-cell differentiation, we examined the expression of co-stimulatory molecules and cytokines critical for plasma cell formation. Mice immunized twice with G+CsA and challenged with RSV exhibited robust upregulation of CD40L in Treg cells within both spleens (Figure 4A and Supplementary Figure S4B) and pulmonary LNs (Supplementary Figure S4C). Similarly, intracellular staining revealed significant IL-10 production by G+CsA-induced Treg cells (Figure 4B, Supplementary Figure S4D,E). These findings suggest that CD40L and IL-10, key mediators of B-cell survival and antibody secretion, are selectively amplified in Treg cells following G+CsA immunization, potentially driving plasma cell differentiation.

To dissect the role of Treg-derived IL-10 in antibody production, we co-cultured Treg cells from G+CsA- or PBS-immunized mice with G protein-primed B cells or PBS-treated B cells, using heat-inactivated RSV as antigen. After 3 days, supernatants from G+CsA-derived Treg co-cultures with G-primed B cells exhibited significantly elevated IL-10 (*p* < 0.05 vs. PBS-derived Treg co-cultures with G-primed B cells) and IgG levels (*p* < 0.01 vs. PBS-derived Treg co-cultures with G-primed B cells; Figure 4C,D). Notably, IL-10 production by G+CsA-induced Tregs correlated with enhanced IgG secretion from antigen-experienced B cells (Figure 4D). To test whether IL-10 and CD40L mediate Treg-dependent B-cell activation, we neutralized IL-10 and blocked CD40L in co-cultures (Figure 4E). This abrogated the ability of G+CsA-induced Tregs to sustain plasma cell differentiation (*p* = 0.0807 vs. isotype control co-culture with G+CsA-induced Teff cells; Figure 4F) and B-cell survival (*p* < 0.01 vs. isotype control co-culture with G+CsA-induced Teff cells; Figure 4G). These data demonstrate that IL-10 and CD40L from G+CsA-induced Tregs are essential for driving antibody production by promoting B-cell survival and differentiation.

To validate these findings in vivo, mice were administered anti-IL-10 or anti-CD40L antibodies intraperitoneally prior to each of two G+CsA immunizations. ELISA analysis revealed that blocking IL-10 or CD40L significantly reduced anti-RSV IgG titers (Figure 4H,I). This indicates that IL-10 and CD40L from G+CsA-induced Treg cells directly contribute to antibody production during immunization. Together, these results establish that Treg-derived IL-10 and CD40L are critical mediators of G+CsA-enhanced humoral immunity.

## 4. Discussion

This study demonstrates that Cyclosporin A (CsA), traditionally an immunosuppressant, functions as a dual-action adjuvant in RSV vaccination by enhancing antibody production via a regulatory T-cell (Treg)-dependent mechanism. In Balb/c mice, G+CsA co-administration dose-dependently increased RSV-specific IgG and neutralizing antibodies, with selective augmentation of IgG1 and IgG2 subclasses. Mechanistically, CsA-induced Tregs expressed CD40L and IL-10, directly promoting B-cell activation, proliferation, and plasma cell differentiation. This represents a novel immunomodulatory pathway distinct from conventional Treg roles, which typically suppress immunity.

The key neutralizing epitopes on the G protein are linear in the central conserved region (CCD). The extensive glycan shield present on expressed G protein in eukaryotic cells can sterically obstruct antibodies. In contrast, employing bacterial expression for the G protein removes glycosylation entirely, making the non-glycosylated G protein an effective target for neutralizing antibodies. CsA’s paradoxical enhancement of immunity reflects its role as an immunoregulator rather than a strict suppressor. Treg cells, critical during RSV infection for maintaining immune homeostasis and preventing excessive inflammation [43,44,45,46], typically suppress immune responses to dampen antiviral clearance. However, in the context of vaccination, Tregs can regulate immunity to prevent overactivation, promoting a balanced response. Our previous work demonstrated that optimal CsA doses (10 μg/animal) induced the best anti-G antibody level accompanied with the lowest T-cell proliferation. Higher doses (20 μg/animal) would cause off-target effects [32]. The CX3C motif in the G protein’s conserved central domain (CCD) contributes to T-cell and inflammatory cell migration. However, immunization with G protein alone induced fewer Tregs and less G-specific IgG than the G + CsA combination. This suggests that Treg induction is driven primarily by the CsA adjuvant. Both the CsA-alone and G-alone groups exhibited significantly fewer Tregs compared to the PBS and G+CsA groups (Figure 3B,C). This decrease may result from Treg cell death and/or expansion of effector T cells, though further investigation is needed to clarify the underlying mechanism.

Our data show that G+CsA-induced Tregs adopt a unique helper phenotype, distinct from conventional Tregs, by upregulating CD40L and IL-10 to drive B-cell antibody production unlike T follicular helper (Tfh) cells or other effector T cells (Th1/Th2/Th17), which conventionally mediate antibody responses. Blocking IL-10 or CD40L abrogated both in vitro B-cell survival and in vivo antibody titers, confirming these cytokines as critical mediators. The in vitro and in vivo systems differ mechanistically due to microenvironmental complexity. In vitro, plasma cells depend on CD40L (T-cell contact) and IL-10 (paracrine signaling) for survival [47,48]. Blocking both pathways disrupts this synergy (Figure 4F). In vivo, however, stromal-derived BAFF/APRIL (via BCMA) and myeloid cell signals compensate for partial pathway inhibition [49,50], allowing single-antibody treatments to retain efficacy (Figure 4H). This redundancy aligns with the microenvironment-dependent plasticity of plasma cell survival described by Tangye and Tarlinton [51]. Our findings suggest CsA could optimize RSV vaccine efficacy by fine-tuning antibody isotypes (IgG1/IgG2) and enhancing humoral immunity. Analysis of Tfh cells at day 4 post-RSV infection after boosting immunization may have preceded peak Tfh accumulation (days 10–14) [52]. Future studies will include longitudinal profiling to capture dynamic Tfh–B-cell interactions.

An interesting phenotype we observed was that the percentage of plasma cells in the G+CsA group was significantly higher than those in both the G-alone and CsA-alone groups (Figure 2E), suggesting that the combination of G protein and CsA effectively promotes the accumulation of mature plasma cells in the spleen for long-term survival [53]. Conversely, the presence of plasma cells in the G+CsA group within the draining lymph nodes (inguinal LNs) was significantly lower than that observed in the CsA-alone and PBS groups and comparable to that in the G-alone group (Figure 2D). This finding suggested that the G protein challenge resulted in the production of short-lived plasma cells in the CsA-alone and PBS groups, whereas the G+CsA and G-alone groups exhibited a reduced generation of such cells. Moreover, the antigen specificity of G+CsA-induced GC B cells and long-lived plasma cells will also be examined in a future study.

While subcutaneous immunization may primarily activate local lymph nodes, our focus on pulmonary lymph nodes reflects their critical role as hubs for RSV infection-related immune responses. The lung is the primary target organ of RSV, and its pulmonary lymph nodes are central to coordinating antiviral immunity and modulating immunopathology. This choice is further supported by evidence that pulmonary LNs exhibit sustained increases in dendritic cell populations and T-cell activation during RSV infection, underscoring their importance in antiviral defense and immunoregulation [54]. Future studies will also evaluate B-cell responses in lymph nodes proximal to the immunization site, such as inguinal or axillary lymph nodes, to provide a more comprehensive understanding of the immune dynamics involved.

We chose activated Tregs from RSV-challenged mice based on our findings prior to infection and antigen challenge, which demonstrated no significant differences in splenic Treg frequencies and IL-10 expression in splenic Treg and CD4+ T cells between the G+CsA and PBS groups (Supplementary Figure S5A,B). With OVA as antigen, we found there was an increased trend in the OVA+CsA group compared with either the OVA-alone or CsA-alone groups, but no significant difference, either in the spleen or in the draining lymph nodes (inguinal LNs) (Supplementary Figure S5D,E). Furthermore, our previous study [32] showed that RSV challenge markedly amplifies G+CsA-induced Treg activation, resulting in elevated IL-10 production and potent suppression of pathogenic T-cell proliferation, thereby limiting vaccine-associated side effects. The pro-B-cell function of Tregs occurs after immunization—particularly after the booster dose—when vaccine-induced Tregs are re-stimulated and activated by antigen. To more faithfully mimic the microenvironment of post-immunization crosstalk between activated Tregs and B cells, we therefore purified RSV-activated Tregs from challenged mice for our in vitro experiments. This approach also allowed us to enrich for antigen-specific Tregs, as unchallenged mice would not provide a sufficient population of these cells. We will continue to explore whether G+CsA-induced Tregs retain B-cell helper capacity under homeostatic conditions in future studies.

The dose-dependent effect of CsA underscores its potential for precise adjuvant dosing, avoiding excessive inflammation while maximizing protection. Moreover, the unique helper function of G+CsA-induced Tregs enhances RSV-specific humoral immunity via IL-10 and CD40L. The increased Treg frequencies in infants compared with those in adults may influence the translational relevance of our findings. While neonatal murine studies indicate CsA primes protective Treg responses without VAERD [31], age-dependent differences in Treg functionality (e.g., Th17/Treg balance in pediatric RSV infection) warrant further investigation. This approach could be extended to other pathogens in which Treg modulation could enhance antibody responses without compromising safety.

Our findings align with emerging reports of combinatorial adjuvant strategies. Han et al. demonstrated that IL-10/type I interferon crosstalk enhances RSV-G vaccine efficacy [55], while Su et al. showed CD40L agonism synergizes with prefusion F-protein immunogens [40]. Together, these studies underscore the therapeutic potential of dual-pathway modulation to amplify humoral immunity. While our murine model provides proof of concept, caution is warranted in extrapolating these findings to humans. Future work should address species-specific immune differences, evaluate long-term immune memory, and assess interactions with other variables affecting immune responses. A more comprehensive analysis of cellular immunity, beyond antibody titers, is also needed.

## 5. Conclusions

In conclusion, this study redefines CsA’s role in vaccination, showcasing its potential to convert an immunosuppressant into an immune enhancer via Treg-mediated B-cell activation. By elucidating the CD40L/IL-10 axis in Treg–B-cell crosstalk, we highlight a promising strategy for RSV vaccine design and beyond. Further investigation into CsA’s adjuvant properties in humans could unlock new opportunities for infectious disease prevention.

## Data Availability

All data reported in this paper will be shared by contacting the lead contact upon request. Any additional information required to reanalyze the data reported in this work is available from the lead contact upon request.

## Supplementary Materials

The following supporting information can be downloaded at https://www.mdpi.com/article/10.3390/vaccines13100997/s1: Figure S1: The gating strategy for GC B cells; Figure S2: Tfh percent and IL-21 expression after immunization and infection; Figure S3: Foxp3 expression in CD4+ T cells from naïve Foxp3-DTR/EGFP mice before (up) and after (down) DT injection were tested by FACS. Figure S4: IL-10 and CD40L were highly expressed in G+CSA-induced Treg cells. Figure S5: Treg percent after immunization. Table S1: G Protein’s quality-control testing results summary.

## References

[1] McMorrow M.L., Moline H.L., Toepfer A.P., Halasa N.B., Schuster J.E., Staat M.A., Williams J.V., Klein E.J., Weinberg G.A., Clopper B.R. (2024). Respiratory Syncytial Virus-Associated Hospitalizations in Children <5 Years: 2016–2022. Pediatrics.

[2] Peng R., Chen C., Chen Q., Zhang Y., Huang R., Zhang Y., Li J. (2024). Global progress in clinical research on human respiratory syncytial virus vaccines. Front. Microbiol..

[3] Rainisch G., Adhikari B., Meltzer M.I., Langley G. (2020). Estimating the impact of multiple immunization products on medically-attended respiratory syncytial virus (RSV) infections in infants. Vaccine.

[4] Bowser D.M., Rowlands K.R., Hariharan D., Gervasio R.M., Buckley L., Halasa-Rappel Y., Glaser E.L., Nelson C.B., Shepard D.S. (2022). Cost of Respiratory Syncytial Virus Infections in US Infants: Systematic Literature Review and Analysis. J. Infect. Dis..

[5] Matias G., Taylor R., Haguinet F., Schuck-Paim C., Lustig R., Shinde V. (2017). Estimates of hospitalization attributable to influenza and RSV in the US during 1997–2009, by age and risk status. BMC Public Health.

[6] Kulkarni H., Smith C.M., Lee Ddo H., Hirst R.A., Easton A.J., O’Callaghan C. (2016). Evidence of Respiratory Syncytial Virus Spread by Aerosol. Time to Revisit Infection Control Strategies?. Am. J. Respir. Crit. Care Med..

[7] Kutter J.S., Spronken M.I., Fraaij P.L., Fouchier R.A., Herfst S. (2018). Transmission routes of respiratory viruses among humans. Curr. Opin. Virol..

[8] Scheltema N.M., Gentile A., Lucion F., Nokes D.J., Munywoki P.K., Madhi S.A., Groome M.J., Cohen C., Moyes J., Thorburn K. (2017). Global respiratory syncytial virus-associated mortality in young children (RSV GOLD): A retrospective case series. Lancet Glob. Health.

[9] Weinberg G.A. (2017). Respiratory syncytial virus mortality among young children. Lancet Glob. Health.

[10] Piedimonte G., Perez M.K. (2014). Respiratory syncytial virus infection and bronchiolitis. Pediatr. Rev..

[11] Sande C.J. (2024). The long-term efficacy of a respiratory syncytial virus vaccine for older adults. Lancet Infect. Dis..

[12] Hammitt L.L., Dagan R., Yuan Y., Cots M.B., Bosheva M., Madhi S.A., Muller W.J., Zar H.J., Brooks D., Grenham A. (2022). Nirsevimab for Prevention of RSV in Healthy Late-Preterm and Term Infants. N. Engl. J. Med..

[13] Mori M., Yoshizaki K., Watabe S., Ishige M., Hinoki A., Kondo T., Taguchi T., Hasegawa H., Hatata T., Tanuma N. (2023). Safety, efficacy and pharmacokinetics of palivizumab in off-label neonates, infants, and young children at risk for serious respiratory syncytial virus infection: A multicenter phase II clinical trial. Lancet Reg. Health West. Pac..

[14] Mazur N.I., Terstappen J., Baral R., Bardají A., Beutels P., Buchholz U.J., Cohen C., Crowe J.E., Cutland C.L., Eckert L. (2023). Respiratory syncytial virus prevention within reach: The vaccine and monoclonal antibody landscape. Lancet Infect. Dis..

[15] Scotta M.C., Stein R.T. (2023). Current strategies and perspectives for active and passive immunization against Respiratory Syncytial Virus in childhood. J. Pediatr..

[16] Madhi Shabir A., Polack Fernando P., Piedra Pedro A., Munoz Flor M., Trenholme Adrian A., Simões Eric A.F., Swamy Geeta K., Agrawal S., Ahmed K., August A. (2020). Respiratory Syncytial Virus Vaccination during Pregnancy and Effects in Infants. N. Engl. J. Med..

[17] Wesley C., Winckworth L.C. (2022). Respiratory syncytial virus vaccination in pregnancy is not effective enough at reducing infant infections. Arch. Dis. Child. Educ. Pract. Ed..

[18] Knudson C.J., Hartwig S.M., Meyerholz D.K., Varga S.M. (2015). RSV Vaccine-Enhanced Disease Is Orchestrated by the Combined Actions of Distinct CD4 T Cell Subsets. PLOS Pathog..

[19] Bigay J., Le Grand R., Martinon F., Maisonnasse P. (2022). Vaccine-associated enhanced disease in humans and animal models: Lessons and challenges for vaccine development. Front. Microbiol..

[20] Acosta Patricio L., Caballero Mauricio T., Polack Fernando P. (2016). Brief History and Characterization of Enhanced Respiratory Syncytial Virus Disease. Clin. Vaccine Immunol..

[21] Christiaansen A.F., Knudson C.J., Weiss K.A., Varga S.M. (2014). The CD4 T cell response to respiratory syncytial virus infection. Immunol. Res..

[22] Rosenberg H.F., Dyer K.D., Domachowske J.B. (2009). Respiratory viruses and eosinophils: Exploring the connections. Antivir. Res..

[23] Habibi M.S., Jozwik A., Makris S., Dunning J., Paras A., DeVincenzo J.P., de Haan C.A.M., Wrammert J., Openshaw P.J.M., Chiu C. (2015). Impaired Antibody-mediated Protection and Defective IgA B-Cell Memory in Experimental Infection of Adults with Respiratory Syncytial Virus. Am. J. Respir. Crit. Care Med..

[24] Qiu X., Xu S., Lu Y., Luo Z., Yan Y., Wang C., Ji J. (2022). Development of mRNA vaccines against respiratory syncytial virus (RSV). Cytokine Growth Factor. Rev..

[25] Jordan E., Jenkins V., Silbernagl G., Chávez M.P.V., Schmidt D., Schnorfeil F., Schultz S., Chen L., Salgado F., Jacquet J.-M. (2024). A multivalent RSV vaccine based on the modified vaccinia Ankara vector shows moderate protection against disease caused by RSV in older adults in a phase 3 clinical study. Vaccine.

[26] Loebbermann J., Durant L., Thornton H., Johansson C., Openshaw P.J. (2013). Defective immunoregulation in RSV vaccine-augmented viral lung disease restored by selective chemoattraction of regulatory T cells. Proc. Natl. Acad. Sci. USA.

[27] Fulton R.B., Meyerholz D.K., Varga S.M. (2010). Foxp3+ CD4 Regulatory T Cells Limit Pulmonary Immunopathology by Modulating the CD8 T Cell Response during Respiratory Syncytial Virus Infection. J. Immunol..

[28] Trujillo-Ochoa J.L., Kazemian M., Afzali B. (2023). The role of transcription factors in shaping regulatory T cell identity. Nat. Rev. Immunol..

[29] Laidlaw B.J., Lu Y., Amezquita R.A., Weinstein J.S., Vander Heiden J.A., Gupta N.T., Kleinstein S.H., Kaech S.M., Craft J. (2017). Interleukin-10 from CD4^+^ follicular regulatory T cells promotes the germinal center response. Sci. Immunol..

[30] León B., Bradley J.E., Lund F.E., Randall T.D., Ballesteros-Tato A. (2014). FoxP3+ regulatory T cells promote influenza-specific Tfh responses by controlling IL-2 availability. Nat. Commun..

[31] Zhang S., Zhao G., Su C., Li C., Zhou X., Zhao W., Zhong Y., He Z., Peng H., Dong A. (2020). Neonatal priming and infancy boosting with a novel respiratory syncytial virus vaccine induces protective immune responses without concomitant respiratory disease upon RSV challenge. Hum. Vaccines Immunother..

[32] Li C., Zhou X., Zhong Y., Li C., Dong A., He Z., Zhang S., Wang B. (2016). A Recombinant G Protein Plus Cyclosporine A-Based Respiratory Syncytial Virus Vaccine Elicits Humoral and Regulatory T Cell Responses against Infection without Vaccine-Enhanced Disease. J. Immunol..

[33] Kang Y., Xu L., Wang B., Chen A., Zheng G. (2008). Cutting edge: Immunosuppressant as adjuvant for tolerogenic immunization. J. Immunol..

[34] Fedechkin S.O., George N.L., Wolff J.T., Kauvar L.M., DuBois R.M. (2018). Structures of respiratory syncytial virus G antigen bound to broadly neutralizing antibodies. Sci. Immunol..

[35] Caidi H., Miao C., Thornburg N.J., Tripp R.A., Anderson L.J., Haynes L.M. (2018). Anti-respiratory syncytial virus (RSV) G monoclonal antibodies reduce lung inflammation and viral lung titers when delivered therapeutically in a BALB/c mouse model. Antivir. Res..

[36] Liu J., Ruckwardt Tracy J., Chen M., Johnson Teresa R., Graham Barney S. (2009). Characterization of Respiratory Syncytial Virus M- and M2-Specific CD4 T Cells in a Murine Model. J. Virol..

[37] Miroux C., Moralès O., Carpentier A., Dharancy S., Conti F., Boleslowski E., Podevin P., Auriault C., Pancré V., Delhem N. (2009). Inhibitory Effects of Cyclosporine on Human Regulatory T Cells In Vitro. Transplant. Proc..

[38] Nowosad C.R., Spillane K.M., Tolar P. (2016). Germinal center B cells recognize antigen through a specialized immune synapse architecture. Nat. Immunol..

[39] Bian L., Zheng Y., Guo X., Li D., Zhou J., Jing L., Chen Y., Lu J., Zhang K., Jiang C. (2022). Intramuscular Inoculation of AS02-Adjuvanted Respiratory Syncytial Virus (RSV) F Subunit Vaccine Shows Better Efficiency and Safety Than Subcutaneous Inoculation in BALB/c Mice. Front. Immunol..

[40] Su C., Zhong Y., Zhao G., Hou J., Zhang S., Wang B. (2022). RSV pre-fusion F protein enhances the G protein antibody and anti-infectious responses. npj Vaccines.

[41] Ersching J., Efeyan A., Mesin L., Jacobsen J.T., Pasqual G., Grabiner B.C., Dominguez-Sola D., Sabatini D.M., Victora G.D. (2017). Germinal Center Selection and Affinity Maturation Require Dynamic Regulation of mTORC1 Kinase. Immunity.

[42] McCarron M.J., Park P.W., Fooksman D.R. (2017). CD138 mediates selection of mature plasma cells by regulating their survival. Blood.

[43] Ruckwardt T.J., Bonaparte K.L., Nason M.C., Graham B.S. (2009). Regulatory T cells promote early influx of CD8+ T cells in the lungs of respiratory syncytial virus-infected mice and diminish immunodominance disparities. J. Virol..

[44] Mangodt T.C., Van Herck M.A., Nullens S., Ramet J., De Dooy J.J., Jorens P.G., De Winter B.Y. (2015). The role of Th17 and Treg responses in the pathogenesis of RSV infection. Pediatr. Res..

[45] van Nieuwenhuijze A., Liston A. (2015). The Molecular Control of Regulatory T Cell Induction. Prog. Mol. Biol. Transl. Sci..

[46] Connors M., Kulkarni A.B., Firestone C.Y., Holmes K.L., Morse H.C., Sotnikov A.V., Murphy B.R. (1992). Pulmonary histopathology induced by respiratory syncytial virus (RSV) challenge of formalin-inactivated RSV-immunized BALB/c mice is abrogated by depletion of CD4+ T cells. J. Virol..

[47] Cassese G., Arce S., Hauser A.E., Lehnert K., Moewes B., Mostarac M., Muehlinghaus G., Szyska M., Radbruch A., Manz R.A. (2003). Plasma Cell Survival Is Mediated by Synergistic Effects of Cytokines and Adhesion-Dependent Signals. J. Immunol..

[48] Bryant V.L., Ma C.S., Avery D.T., Li Y., Good K.L., Corcoran L.M., de Waal Malefyt R., Tangye S.G. (2007). Cytokine-Mediated Regulation of Human B Cell Differentiation into Ig-Secreting Cells: Predominant Role of IL-21 Produced by CXCR5+ T Follicular Helper Cells. J. Immunol..

[49] Mackay F., Schneider P. (2009). Cracking the BAFF code. Nat. Rev. Immunol..

[50] O’Connor B.P., Raman V.S., Erickson L.D., Cook W.J., Weaver L.K., Ahonen C., Lin L.-L., Mantchev G.T., Bram R.J., Noelle R.J. (2004). BCMA Is Essential for the Survival of Long-lived Bone Marrow Plasma Cells. J. Exp. Med..

[51] Tangye S.G., Tarlinton D.M. (2009). Memory B cells: Effectors of long-lived immune responses. Eur. J. Immunol..

[52] Laidlaw B.J., Cyster J.G. (2021). Transcriptional regulation of memory B cell differentiation. Nat. Rev. Immunol..

[53] Fooksman D.R., Jing Z., Park R. (2024). New insights into the ontogeny, diversity, maturation and survival of long-lived plasma cells. Nat. Rev. Immunol..

[54] Smit J.J., Rudd B.D., Lukacs N.W. (2006). Plasmacytoid dendritic cells inhibit pulmonary immunopathology and promote clearance of respiratory syncytial virus. J. Exp. Med..

[55] Han R., Wang T., Cheng X., Bing J., Li J., Deng Y., Shan X., Zhang X., Wang D., Sun S. (2024). Immune Responses and Protection Profiles in Mice Induced by Subunit Vaccine Candidates Based on the Extracellular Domain Antigen of Respiratory Syncytial Virus G Protein Combined with Different Adjuvants. Vaccines.

