# Supplementary material for: Dual-Function Adjuvant Cyclosporin A: Enhancing RSV-Specific Humoral Immunity via Treg-Driven B-Cell Activation
Source: Vaccines (Basel). 2025 Sep 23;13(10):997. doi: 10.3390/vaccines13100997 (PMC12567732; doi:10.3390/vaccines13100997)
Supplement: Supplementary file 1 [file vaccines-13-00997-s001.zip › vaccines-3841935-supplementary.pdf]

SUPPLEMENTARY DATA

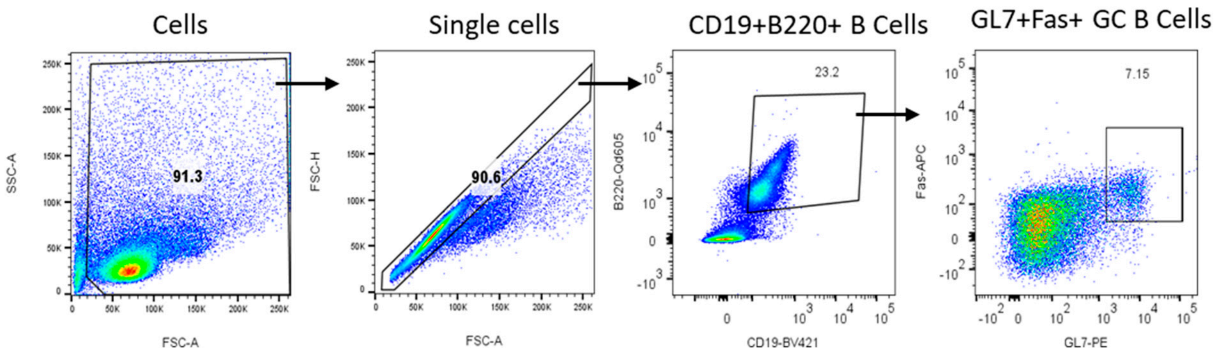

Supplementary Figure S1. The gating strategy for GC B cells.

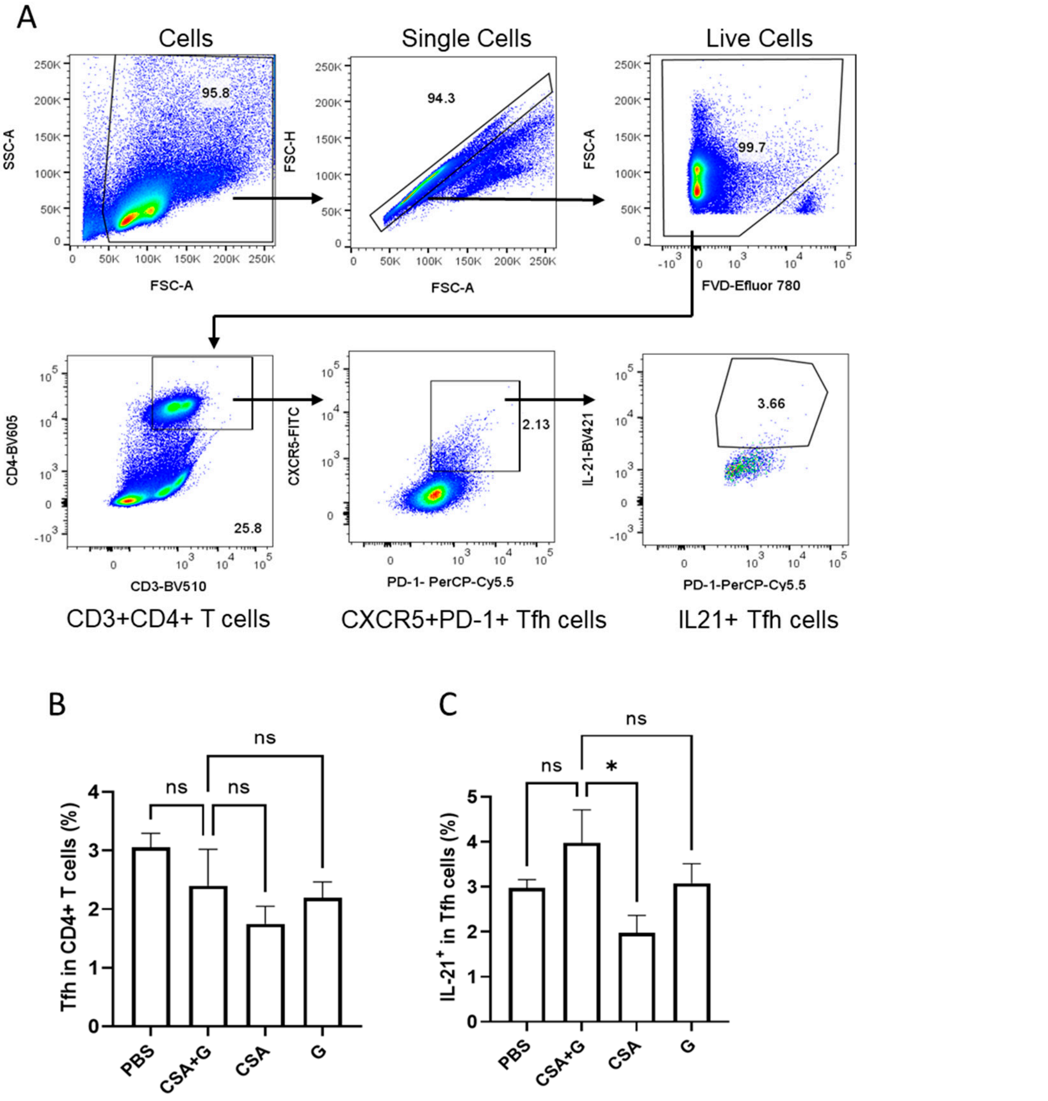

Supplementary Figure S2. Tfh percent and IL-21 expression after immunization and infection. According to the experiment schematic shown in Figure. 3A. **(A)** the gating strategy for Tfh cells. **(B)** The ratio of Tfh cells from spleen on Day 32 after immunization and RSV infection. **(C)** IL-21 secreting Tfh cells from spleen on Day 32 after vaccine immunization and RSV infection. Data shown are means ± SEM from two independent experiments (n=5). **p < 0.001, **p < 0.01, *p < 0.05, ns (not significant)were determined by One-way ANOVA (A, B).

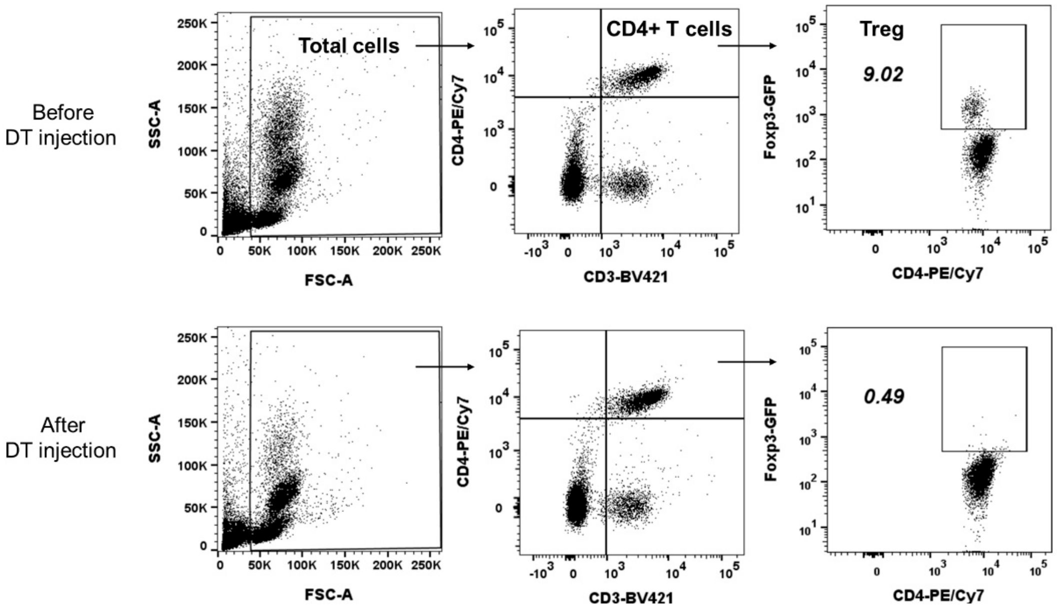

Supplementary Figure S3. Foxp3 expression in CD4[+] T cells from naïve Foxp3-DTR/EGFP mice before **(up)** and after **(down)** DT injection were tested by FACS. Foxp3-DTR/EGFP mice were injected i.p. with 500 ng DT in 100 µl PBS on days -2 and -1. Blood samples were collected before DT injection and on day 0 after the two DT injections. The Foxp3 expression in CD4[+] T cells in blood were tested.

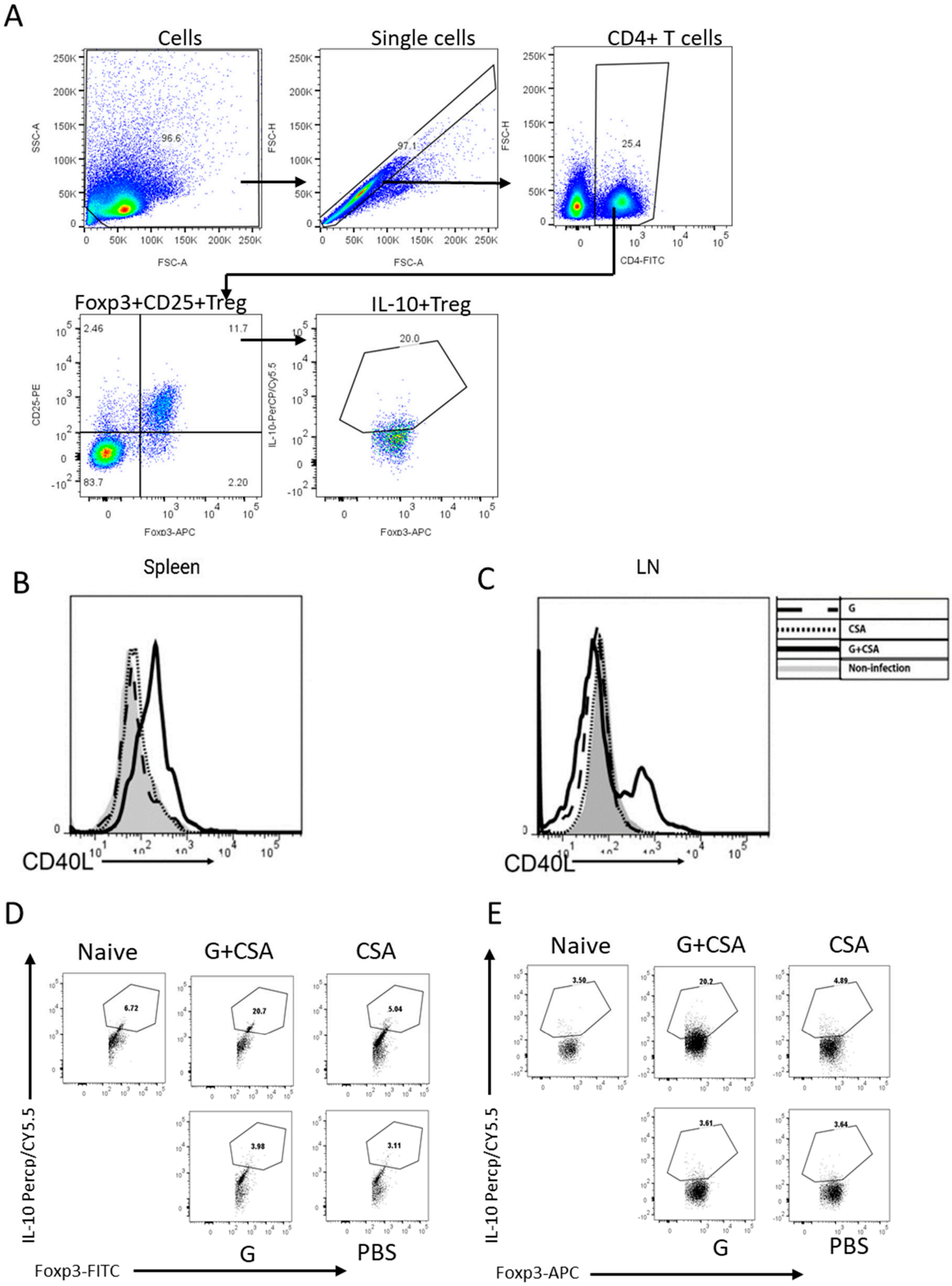

Supplementary Figure S4. IL-10 and CD40L high expressed in G+CSA induced Treg cells. FACS gate stratepy for CD4+Foxp3+Treg was shown **(A)**. Histogram of CD40L expression in the Treg cells after RSV infection in the spleens **(B)** and pulmonary LNs **(C).** Dot plots of IL-10 secreting Treg cells in the spleens **(D)** and pulmonary LNs **(E)** after RSV infection.

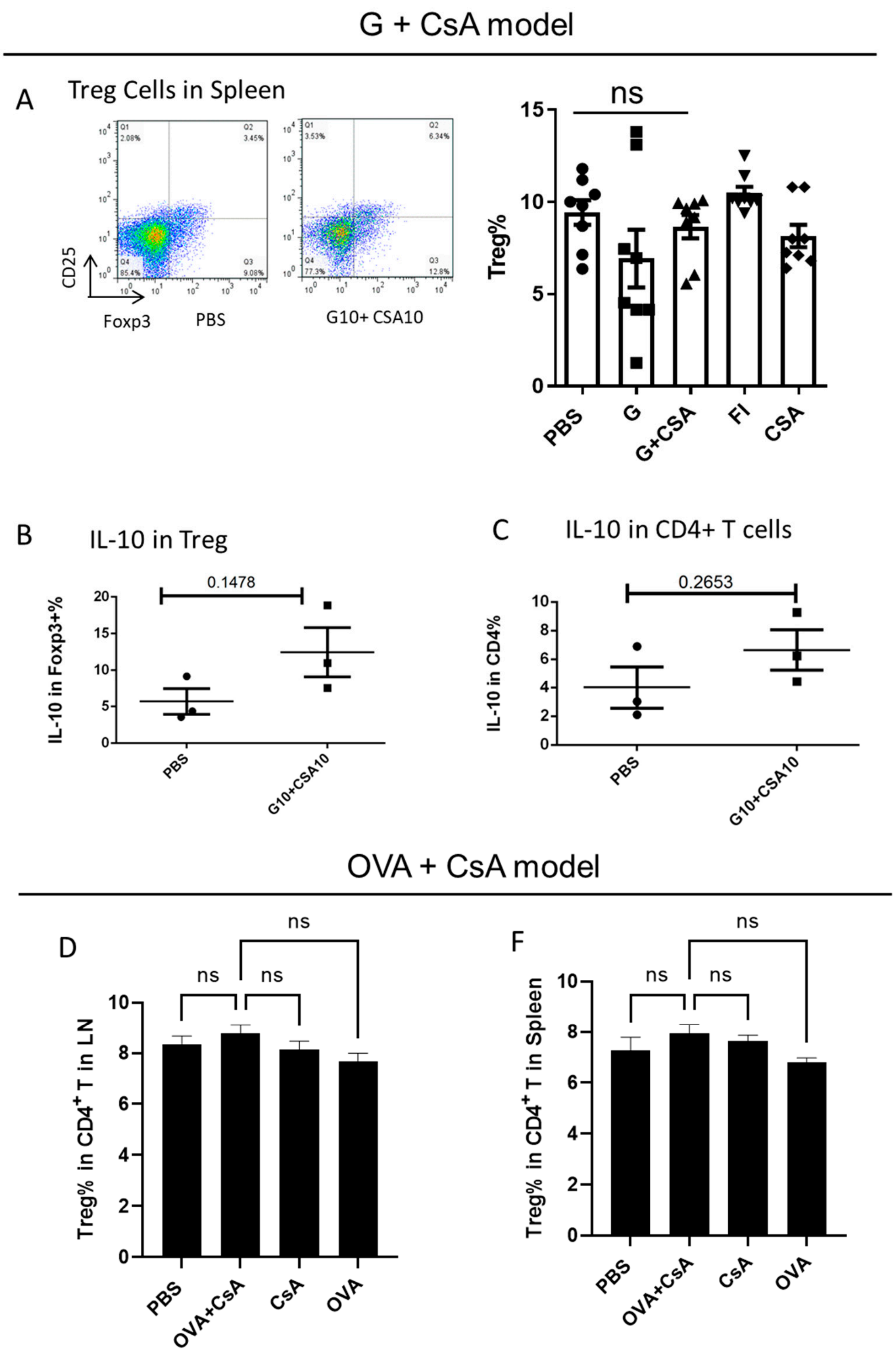

Supplementary Figure S5. Treg percent after immunization. In G protein plus CsA model, Balb/c mice were immunized s.c. on days 0 and 14 with 10μg G + 10μg CsA, 10μg G alone, 10μg CsA alone, FI-RSV, or PBS, and splenic cells was tested on day 28 prior to infection. (**A**) The histogram and statistical graph presented the percentage of CD25+Foxp3+ Treg cells in the spleen. Data shown are means ± SEM pooled from two independent experiments (n=4). ns (not significant)were determined by One-way ANOVA. The expression of IL-10 in Tregs **(B)** and CD4+ T cells **(C)** was also assessed. Data shown are means ± SEM from two independent experiments (n=3). In OVA protein plus CsA model, Balb/c mice were immunized s.c. on days 0 and 14 with 10μg OVA + 10μg CsA, 10μg CsA, 10μg OVA, or PBS, and Foxp3+CD25+ Treg cells was tested on day 28 in **(D)** inguinal LNs and **(E)** spleens. Data shown are means ± SEM from two independent experiments (n=7). ns (not significant)were determined by One-way ANOVA (D, E).

Supplementary Table S1. G Protein 's Quality Control Testing Results Summary.

Test Items	Acceptance Criteria	Test Results
Purity	Not less than 90%	>99%
Bacterial Endotoxins	Less than 10 EU per mL	< 5 EU/mL
Sterility	Comply with Test for Sterility	No bacteria growth
